# Postcommercialisation outcomes of bridge‐enhanced anterior cruciate ligament restoration: The first 100 Bridge registry patients

**DOI:** 10.1002/ksa.12806

**Published:** 2025-08-13

**Authors:** Jocelyn Wittstein, Sabrina Strickland, Andreas Gomoll, Seth Sherman, Brian Lau, Dean Taylor, Dennis Kramer, Sean Keyes, Alexander K. Meininger, Marc Pietropaoli, Sean McMillan, Daryl Osbahr, Jacqueline Brady

**Affiliations:** ^1^ Department of Orthopaedic Surgery Duke University School of Medicine Durham North Carolina USA; ^2^ Sports Medicine Institute Hospital for Special Surgery New York New York USA; ^3^ Stanford University Redwood City California USA; ^4^ Boston Children's Hospital, Harvard Medical School Boston Massachusetts USA; ^5^ AdventHealth Orlando Florida USA; ^6^ Steamboat Orthopaedic & Spine Institute Steamboat Springs Colorado USA; ^7^ Victory in Motion Skaneateles New York USA; ^8^ Virtua Health Marlton New Jersey USA; ^9^ Rothman Orthopaedics Florida at AdventHealth Orlando Florida USA; ^10^ Oregon Health & Science University Portland Oregon USA

**Keywords:** ACL reconstruction, ACL repair, ACL retear, BEAR, knee

## Abstract

**Purpose:**

To review adverse events and outcomes at least 1 year postoperatively from Bridge enhanced ACL restoration (BEAR) in the first 100 subjects of the Bridge registry, a postcommercialisation prospective cohort.

**Methods:**

Consecutive BEAR patients were invited to enroll in the Bridge registry. Technique modifications, adverse events and reoperations were recorded. Physical examinations and patient reported outcomes (International Knee Documentation Committee [IKDC], knee injury and osteoarthritis outcome score [KOOS], Marx and return to sport index [RSI]) were reported at 0, 6, 9 and 12 months.

**Results:**

Of the 100 subjects, 94 enrolled postoperatively. Four exited the study. Mean follow up was 15.3 ± 4.7 months. The mean age was 31.3 ± 14.3 years. 67% were female. Classic and modified techniques were each used in 50 cases. The rates of adverse events and reoperations at 1 year were: one deep venous thrombosis, two meniscal injuries without anterior cruciate ligament (ACL) retear and eight reoperations (one debridement, two manipulations, three suture removals, one partial lateral meniscectomy and one medial meniscus repair). Adverse events between 1 and 2 years included one recurrent ACL tear with associated meniscus tears, two meniscal injuries without ACL tear, and one isolated ACL tear. Overall reoperation rate was 8.3% (8/96) at 1 year and 11.5% (11/96) at final follow‐up, with 0% ACL retear at 1 year and 2.1% (2/96) ACL retear at final follow‐up. Mean IKDC, Marx and RSI scores at 12 months were (*N* = 79), respectively 82.7 ± 14.6, 7.5 ± 5.36 and 66.6 ± 24.06. Mean KOOS pain, function daily living and function sports and recreational activities were: 92.7 ± 8.6, 96.7 ± 6.0 and 82.0 ± 18.5. Lachman (*N* = 75) was 0–2 mm in 80.0% (60/75) and 3–5 mm in 20.0% (15/75). Pivot shift (*N* = 73) was negative in 90.4% (66/73), grade 1 in 8.2% (6/73) and grade 2 in 1.4% (1/73) at 12 months.

**Conclusion:**

The rates of adverse events of BEAR with and without modification are low at 1 year. Excellent stability and ROM are attained at 1 year.

**Clinical Relevance:**

BEAR has promising results in postcommercial utilization and is as an alternative to reconstruction.

**Level of Evidence:**

Level II cohort study.

**Abbreviations**
ACL: anterior cruciate ligament
BEAR: Bridge enhanced ACL reconstruction
CI: confidence intervals
IKDC: International Knee Documentation Committee
KOOS: knee injury and osteoarthritis outcome score
ROM: range of motion
RSI: return to sport index
VAS: visual analogue scale

## INTRODUCTION

Anterior cruciate ligament (ACL) injuries are common knee injuries, with an annual reported incidence of 68.6 per 100,000 person‐years for isolated ACL tears, with approximately 120,000 ACL injuries occurring annually in the United States, with increasing rates of reconstruction over time [8, 26]. The current operative gold standard treatment for ACL ruptures in young, active patients is autograft ACL reconstruction [18, 21].

ACL reconstruction became the standard treatment after poor results at long‐term follow‐up were reported for primary open ACL repair, including pain, stiffness and an approximately 50% failure rate [4, 21, 23, 28]. Interestingly, among patients who did not require revision stabilisation and had good outcomes after open ACL repair, those good outcomes persisted at 30 years [28]. Sherman et al. additionally shed light on differences in outcomes for different tear locations and demonstrated better outcomes in more proximal tears [27]. Despite findings that ACL reconstruction has lesser long‐term risk of reinjury and revision ACL surgery than repair even with modern‐day techniques, limitations of reconstruction include graft harvest‐related complications, knee pain and an extensive rehabilitation period [1, 2, 9, 18, 21, 22, 23, 25, 31]. Thus, interest remains in optimising outcomes of ACL repair with advances in technology and assessment of risk for reinjury to aid in patient selection.

Recent innovations in surgical techniques and an improved understanding of the mechanisms underlying healing of the ligament have led to a renewed interest in arthroscopic ACL repair [2, 6, 9, 12, 18, 21, 22, 23, 32]. Systematic reviews with meta‐analyses reported similar patient‐reported outcomes for ACL repair compared to ACL reconstruction [2, 9, 18, 21, 22, 32]. ACL repair may be an alternative to ACL reconstruction despite higher retear rates in young patients considering the benefits of less invasive surgical techniques, avoidance of donor site morbidity and harvesting complications and faster postoperative recovery [1, 2, 9, 18, 21, 22, 23, 25, 31, 32]. A new surgical technique is the Bridge‐enhanced ACL restoration (BEAR), which was developed to stimulate the natural healing of the ligament while protecting it from degradation by synovial enzymes. The technique combines suture repair of the ligament and a bioactive scaffold implant to Bridge the gap between the torn ligament ends [12].

Preclinical porcine and canine research using the BEAR technique showed improved healing of the ACL and a significant decrease in the incidence of posttraumatic osteoarthritis [7, 10, 14, 15, 16, 17, 30]. The BEAR technique avoids the need to harvest a graft and preclinical models showed equivalent mechanical results to ACL reconstruction [20, 30]. Clinical data have been collected in three clinical trials (BEAR I, BEAR II and BEAR III) between February 2015 and January 2019. BEAR I was an early feasibility study and showed similar outcomes at the 2‐year follow‐up for BEAR compared to ACL reconstruction with hamstring autograft [13]. BEAR II was a randomised control trial showing noninferiority of BEAR compared to hamstring autograft ACL reconstruction for IKDC and AP translation at 2 years follow‐up with a 14% retear rate in young patients [11]. BEAR III is an ongoing cohort study designed to determine whether age is a risk factor for a worse outcome following the BEAR procedure. Initial results of the BEAR III study combined with results of the BEAR I and II included 26.3% retear in patients under 16 years, 22.5% retear in patients 16–17 years, 12.1% retear in patients age 18–22, and 0% retear in patients over 22 years, with a combined retear rate of 14.6% [24].

Following guidance of the Food and Drug Administration on the collection of Real‐World Data, the Bridge registry study was designed to evaluate the continued safety and performance of the BEAR device in real‐world setting [3]. This is an ongoing multicenter study, and the current manuscript reports the preliminary results on the first 100 patients included in the registry. Performance and safety outcomes are described for the first year following surgery and at final follow‐up for this cohort including reinjury and reoperation rates, surgical complications, physical examination findings and patient‐reported outcome measures. Additionally, impact of surgical technique modifications on outcomes are described. The purpose of this study is to describe rates of adverse events and outcomes in postcommercialisation use of the BEAR procedure.

## MATERIALS AND METHODS

This manuscript describes the preliminary results of the first 100 patients included in an ongoing postmarket registry study (‘the Bridge registry’, NCT05398341) evaluating outcomes of the BEAR procedure. The study is being conducted in the Department of Orthopaedic Surgery at multiple United States study sites and is sponsored by Miach Orthopaedics, Inc. The Bridge registry was approved by a central IRB and the institutional review board at each participating site and all participants granted their informed consent before being included in the registry. The registry started on 28 February 2023 and estimated study completion is January 2027.

### Participants

The study consists of a mixed prospective and retrospective cohort. Patients scheduled for ACL surgery with the BEAR implant, who are willing to provide consent, and who can complete required follow‐up visits and assessments are included in the prospective cohort. Since the registry aims to capture all uses of the implant and associated outcomes, patients who were treated with a BEAR implant prior to initiation of the registry at each participating site were also included retrospectively by enrolling at the time of the first postoperative visit following registry initiation. For the retrospective cohort, eligible patients signed consents after the BEAR surgery was performed and subsequent data was the prospectively collected. Patients with a known allergy to bovine collagen, bovine gelatin or other bovine‐derived products contraindicated in the BEAR implant labelling were not eligible to undergo the BEAR procedure and were excluded. Recommendation for the BEAR procedure was at the discretion of the treating surgeon and the result of a shared decision‐making process with the patient. Strict adherence to recommendations to perform the BEAR procedure within 45 days of injury was not an exclusion criteria, such that days from injury to procedure was not an exclusion criteria.

### Surgical procedure/BEAR implant

The BEAR implant is used as a commercially available product in this study. The BEAR procedure was indicated and performed at the discretion of the treating surgeons with or without deviations from the originally described BEAR procedure technique [12]. In the originally described technique, the ACL repair with the BEAR implant is achieved by placing Vicryl (Ethicon) suture in the native ligament to redirect it to its origin. The repair suture is passed through the base of the stump then in a Bunnell fashion with three passes of each suture tail utilizing a self retrieving arthroscopic suture passer. The repair is initially supported using a bracing suture (Ethibond; Ethicon) that is incorporated into the femoral fixation (a cortical button) and secured to the tibia through a small tunnel placed within the anterior aspect of the ACL tibial footprint. Before the ACL sutures are pulled to the femoral origin, the BEAR implant is loaded onto the bracing sutures, saturated with the patient's own blood and sent along the bracing suture into the intercondylar notch. The ACL sutures are tied over the femoral button and the bracing suture is secured to the tibia by tying over a tibial button. The implant softens and becomes a hydrogel over the torn portion of the ligament while it heals, stabilising the fibrin clot in the gap between the torn ligament ends. The implant is replaced within 6–8 weeks with ACL cells and native collagen and blood vessels consistent with fibrovascular repair tissue [19].

Modifications in the surgical technique from the classic technique included in this series of 100 subjects are as follows: use of an anchor on the femoral wall or tibia, use of luggage tag style suture rather than Bunnell suturing, use of nonabsorbable instead of Vicryl sutures in the ACL stump, or use of suture other than Ethibond as the bracing suture. Suture anchor fixation for the BEAR procedure has been previously described and involves suturing of the ACL stump, then securing these sutures to the lateral wall of the notch with an anchor that is preloaded with additional suture tails to act as the bracing sutures. The ACL stump is tensioned to the anchor prior to passage of the BEAR implant into the notch at the time of passing of the bracing sutures into the tibia [5].

### Assessments and data collection

Consecutive patients with ACL injuries indicated for BEAR at six institutions were invited to enroll in the Bridge registry either preoperatively or at the time of their first postoperative visit after study initiation. Subjects were prospectively followed at 6, 9 and 12, and 24 months after surgery with surveys and at 2 and 6 weeks, 3 months, 6 months and 12 months with physical examination. A phone call follow‐up was completed for those subjects who had reached 24 months postop at time of final data collection. Adverse events including recurrent ACL injury, ligamentous or chondral injuries, subsequent meniscus tears, deep venous thrombosis and reoperations were recorded.

Patients are screened and consented during their initial visit to the study site. After consenting, the following data are collected: demographic information (age, sex, height, weight, BMI, ethnicity and race), time from injury to surgery, medical history including major medical conditions and prior surgeries, patient‐reported questionnaires, pain medication use and physical examination data on both knees. During surgery, procedural details are recorded according to standard of care at the site. This includes a physical exam performed under anaesthesia, arthroscopic examination of the menisci, chondral surfaces, and ACL, location and completeness of ACL tear, and a verification that the ACL stump is sufficient to perform the BEAR procedure.

Assessments consist of adverse event screening, a ‘current status’ questionnaire, knee injury questionnaires and a physical exam. The ‘current status’ questionnaire is completed at every visit and includes pain medication use (e.g., opioids), visual analogue scale (VAS) for pain and return to activity (e.g., work, walking, driving and sport). The knee injury questionnaires completed at baseline and postoperatively include the International Knee Documentation Committee (IKDC) subjective knee evaluation, Marx activity, the knee injury and osteoarthritis outcome score (KOOS) and the return to sport index (RSI). The physical exam consists of evaluating for effusion (absent, small, moderate and large), active and passive flexion and extension range of motion (ROM) measured by visual estimate or goniometer, Lachman and the pivot shift exam. Effusion and ROM are performed at every in‐office visit, while Lachman is done at baseline, surgery and from 3 months postsurgery. The pivot shift exam is performed at baseline, surgery and from 6 months postsurgery.

### Statistical analysis

This registry study is descriptive in nature and no formal hypothesis testing is performed. For continuous variables, descriptive statistics include the mean, standard deviation and the associated 95% confidence intervals (CIs). Categorical variables are summarised using frequency and percentage. Descriptive statistics are presented for the complete population (*n* = 100) and for separate subgroups defined based on surgical technique (classic vs. modified).

Adverse events were compared between subjects whose BEAR procedure was performed using the original classic technique versus modified surgical techniques using Fisher's Exact test. ROM, Lachman grade, firmness of endpoint and pivot shift examinations were reported at 12 months. ROM was compared between baseline and 12 months using a paired *t*‐test. Continuous patient‐reported outcomes (IKDC, KOOS, Marx, RSI) were compared between subjects at baseline, 6 months, 9 months and 12 months using analysis of variance (ANOVA).

## RESULTS

**Figure 1:**
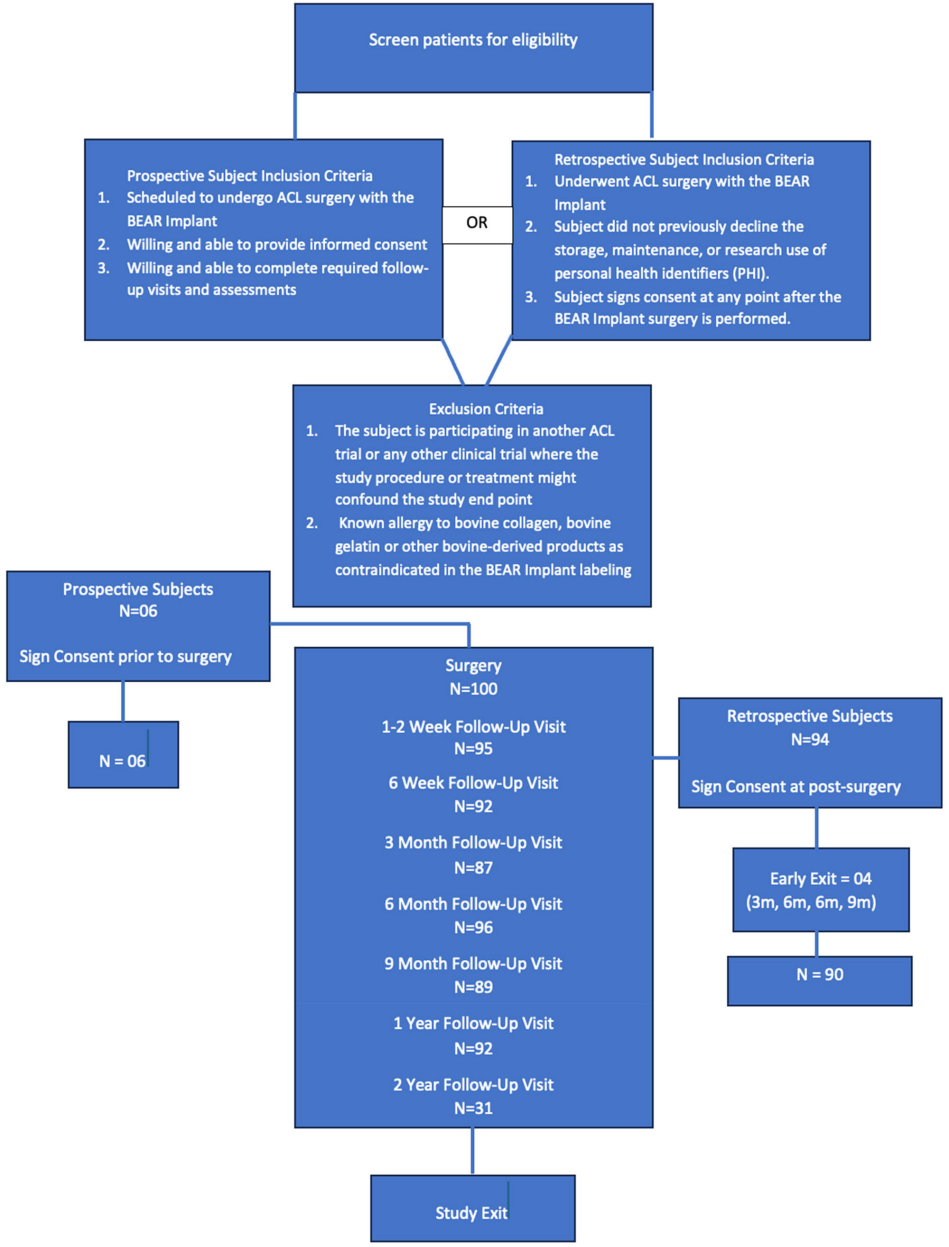
CONSORT flow diagram.

The first 100 patients in the Bridge registry who were at least 1 year postop were included. The first visit of the first patient was on 28 February 2023, the 1‐year visit for the 100th patient was on 31 July 2024. Figure 1 shows the flow of participants in this study. Of the 100 subjects, 94 enrolled postoperatively and 6 enrolled preoperatively. Four exited the study, and one completed the 9‐month, but not the 12‐month follow‐up, providing 95% minimum 1‐year follow‐up (mean 15.5 ± 5.28 months). Thirty‐one of the 100 subjects completed the 2‐year follow‐up.

Demographic information and mean time from injury to surgery are summarised in Table 1. Two‐thirds (67/100) were female and one‐fourth (25/100) were paediatric. The majority of injuries were sports related (77%; 77/100) and noncontact (86%; 86/100). Most injuries were proximal tears (63%; 63/100), while 36% (36/100) were midsubstance and only one injury was distal. Fifty subjects (50%) underwent a BEAR using the classic technique (femoral and tibial buttons, vicryl suture in Bunnell pattern in tibial stump, and ethibond bridging suture), while 50 (50%) subjects underwent a BEAR with a modified surgical technique, meaning some aspect of the procedure was modified from the described classic BEAR technique. All possible modifications and their frequencies are included in Table 2. Suspensory cortical buttons were mainly used for femoral fixation and tibial fixation, with interference‐type anchor fixation being the most common modification. Ethibond was most commonly used to temporarily stabilise the BEAR implant with Suture Tape (Arthrex) being the most common modification. The main suture technique for securing the ACL remnant was the Bunnell technique, with luggage tag sutures being the most common modification. Vicryl was used for the ACL stump suture in most cases, while nonabsorbable suture was used in the other cases. Demographic differences between the classic and modified technique groups are summarised in Table 3, with significant differences in age and sex distribution between the groups. The modified technique group was older and included more female subjects.

**Table 1:** Subject demographics.

	*N* = 100
Study cohort % (*n*/*N*)	
Retrospective enrollment	94.0% (94/100)
Prospective enrollment	6.0% (6/100)
Age (years)^a^	
Mean ± SD (*N*)	31.8 ± 14.3 (100)
Age group % (*n*/*N*)	
<18	25.0% (25/100)
≥18	75.0% (75/100)
≥40	33.0% (33/100)
Paediatric age group % (*n*/*N*)	
<12	8.0% (2/25)
12–13	28.0% (7/25)
14–<18	64.0% (16/25)
Sex % (*n*/*N*)	
Female	67.0% (67/100)
Male	33.0% (33/100)
Ethnicity	
Not Hispanic or Latino	85.6% (83/97)
Hispanic or Latino	14.4% (14/97)
Race % (*n*/*N*)^b^	
White	75.0% (75/100)
Black	9.0% (9/100)
Asian	8.0% (8/100)
American Indian or Alaskan Native	2.0% (2/100)
Other	6.0% (6/100)
Refused	3.0% (3/100)
Unknown	2.0% (2/100)
Height (cm)	
Mean ± SD (*N*)	167.0 ± 11.0 (100)
Weight (kg)	
Mean ± SD (*N*)	71.2 ± 16.0 (100)
Body mass index (kg/m²)	
Mean ± SD (*N*)	25.4 ± 5.0 (100)
Time from injury to surgery (days)	
Mean ± SD (*N*)	45.5 ± 27.1 (100)
Tear type	
Complete	92.0% (92/100)
Partial	8.0% (8/100)

^a^ Age = (date of surgery ‐ date of birth)/365.25.

^b^ Patients were asked to select all that apply, so total number of responses may be greater than number of patients.

**Table 2:** Surgical details.

	*N* = 100
Fixation device used for femoral fixation	
Button	82.0% (82/100)
Anchor	17.0% (17/100)
Other	1.0% (1/100)
Fixation device used for tibial fixation	
Button	57.0% (57/100)
Anchor	43.0% (43/100)
Device used to suture stump	
#2 Vicryl	84.0% (84/100)
Suture tape	12.0% (12/100)
Other	4.0% (4/100)
Device used to suture through BEAR implant	
#2 Ethibond	61.0% (61/100)
Suture tape	30.0% (30/100)
Other	9.0% (9/100)
Suture technique for tibial fixation	
Bunnell	87.0% (87/100)
Luggage tag	13.0% (13/100)
Surgical technique	
Classic	50.0% (50/100)
Modified	50.0% (50/100)

**Table 3:** Subject age and gender by surgical technique.

	Classic technique *N* = 50	Modified technique *N* = 50	*p* value^a^
Age (years)^b^			<0.001
Mean ± SD (*N*)	26.2 ± 14.5 (50)	37.5 ± 11.7 (50)	
Gender % (*n*/*N*)			0.019
Female	56.0% (28/50)	78.0% (39/50)	
Male	44.0% (22/50)	22.0% (11/50)	

^a^ *p* value for age determined from two‐sided two sample *t*‐test. *p*‐Value for gender from chi‐square test.

^b^ Age = (date of surgery ‐ date of birth)/365.25.

### Physical examination results

There was no significant loss of extension as compared to baseline. The mean amount of passive hyperextension at 12 months was 1.6 ± 4.1 degrees (*N* = 69). There was significant improvement in passive flexion with mean flexion at baseline 114.2 ± 31.6 degrees (*N* = 85) and mean flexion at 12 months 136 ± 18.5 degrees (*N* = 73) (*p* < 0.001).

Effusions were uncommon at 12 months postop with 87.1% (61/70) demonstrating no effusion, 7.1% (5/70) having a trace effusion, and 5.7% (4/70) having a moderate effusion.

Lachman's (*N* = 75) was 0–2 mm in 80.0% (60/75) and 3–5 mm in 20.0% (15/75) with 93.8% (61/65) having a firm endpoint (*N* = 65). Pivot shift (*N* = 73) was negative in 90.4% (66/73) and grade 1 in 8.2% (6/73), while 1.4% (1/73) had a grade 2 pivot shift at 12 months.

### Adverse events

**Figure 2:**
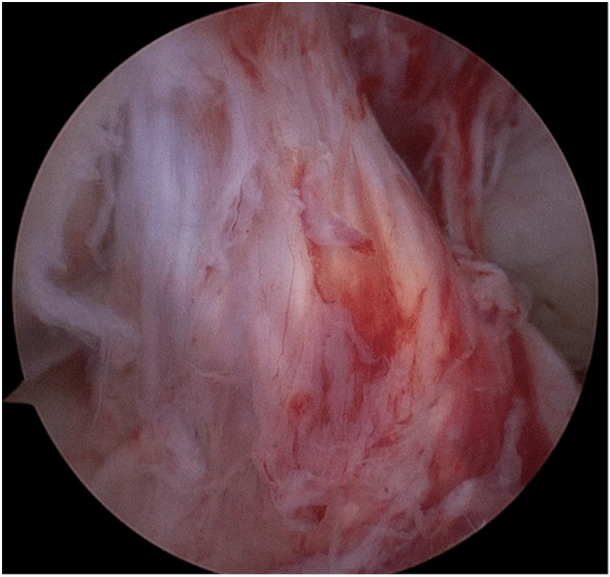
Intraoperative image from arthroscopic lysis of adhesions demonstrating bridging tissue from the femoral to tibial attachment sites.

The overall rates of specific adverse events and reoperations are as follows at 1 year: 1 DVT, two meniscal injuries without ACL retear and eight reoperations (one lysis of adhesions/cyclops removal, two manipulations under anaesthesia, three removals of retained Ethibond suture, one partial lateral menisectomy and one medial meniscus repair). Intraoperative image from arthroscopic lysis of adhesions is provided in Figure 2 demonstrating bridging tissue from the femoral to tibial attachment sites. Adhesions are visible anterior to the native ACL insertion, consistent with slightly anterior placement of the stuture tunnel and BEAR implant per originally described technique.

Adverse events requiring reoperations between 1 and 2 years were one recurrent ACL tear with associated medial and lateral meniscus tears and two meniscal injuries without ACL tear. An additional ACL retear occurred between 1 and 2 years, but has not yet been reoperated on. Overall reoperation rate was 8.3% (8/96) at 1 year and 11.5% (11/96) at final follow‐up, with 0% (0/96) retear rate at 1 year and 2.1% (2/96) ACL retear rate in this whole cohort at final follow‐up. There was also one contralateral ACL tear between 1 and 2 years. There were no significant differences in adverse events or reoperation rates between the classic technique and the modified technique groups. Table 4 summarises adverse events and reoperations in the classic versus modified technique groups.

**Table 4:** Retears, meniscus injuries and additional surgical interventions within 2 years of surgery by surgical technique.

	Within 1 year			Within 2 years		
	Number of events	Number of subjects with events	*p* value	Number of events	Number of subjects with events	*p* value^a^
Classic technique						
Retears	0	0.0% (0/48)		1	4.5% (1/22)	>0.999
With meniscus injury	0	0.0% (0/48)		1	4.5% (1/22)	
Contralateral tears	0	0.0% (0/48)		1	4.5% (1/22)	>0.999
Meniscus injury without anterior cruciate ligament (ACL) retear	1	2.1% (1/48)	>0.999	3	13.6% (3/22)	0.617
Medial meniscus	1	2.1% (1/48)		1	4.5% (1/22)	
Lateral meniscus	0	0.0% (0/48)		1	4.5% (1/22)	
Medial and lateral meniscus	0	0.0% (0/48)		1	4.5% (1/22)	
Other additional surgeries	5	10.4% (5/48)	0.204	6	27.3% (6/22)	0.112
Removal of ethibond nonabsorbable suture	3	6.3% (3/48)		4	18.2% (4/22)	
Knee arthroscopy w lysis of adhesions	1	2.1% (1/48)		1	4.5% (1/22)	
MUA	1	2.1% (1/48)		1	4.5% (1/22)	
Modified technique						
Retears	0	0.0% (0/48)		0	0.0% (0/9)	>0.999
With meniscus injury	0	0.0% (0/48)		0	0.0% (0/9)	
Contralateral Tears	0	0.0% (0/48)		0	0.0% (0/9)	>0.999
Meniscus injury without ACL retear	1	2.1% (1/48)	>0.999	1	11.1% (1/9)	0.617
Medial meniscus	0	0.0% (0/48)		0	0.0% (0/9)	
Lateral meniscus	1	2.1% (1/48)		1	11.1% (1/9)	
Medial and lateral meniscus	0	0.0% (0/48)		0	0.0% (0/9)	
Other additional surgeries	1	2.1% (1/48)	0.204	1	11.1% (1/9)	0.112
R knee manipulation under anaesthesia	1	2.1% (1/48)		1	11.1% (1/9)	

*Note*: Denominators consist of subjects who reached the indicated time point or experienced any listed event prior to it.

^a^ *p* values from Fisher's Exact test comparing the specified complication between surgical techniques at each time point.

### Current status and knee injury questionnaire results

Overall, results on the patient‐reported knee injury questionnaires showed an improvement over time from baseline to 1 year. Results of the knee injury questionnaires are provided in Table 5 All the KOOS subscale scores and IKDC improved significantly from baseline to 12 months (*p* < 0.05). The Marx activity score was statistically insignificantly lower at 12 months than at baseline, but with only 36 subjects to calculate the change score due to low preoperative completion rates (n.s). RSI significantly improved from 6 to 12 months postop (*p* < 0.05). At 12 months 68% of subjects reported return to play.

**Table 5:** Results of current status and knee injury questionnaires for the first 100 Bridge registry patients.

	Baseline	6 months	9 months	1 year	Change from baseline to 1 year mean	ANOVA *p* value
IKDC knee evaluation						
Mean ± SD (*N*)	44.7 ± 15.9 (47)	69.2 ± 16.9 (72)	78.4 ± 15.3 (73)	82.7 ± 14.6 (79)	39.4 ± 2 (*N* = 43)	<0.001
95% CI of mean	(40.0, 49.3)	(65.3, 73.2)	(74.8, 82.0)	(79.4, 85.9)	(33.9, 44.9)	
Marx (total)						
Mean ± SD (*N*)	8.4 ± 5.26 (39)	8.9 ± 5.46 (63)	7.0 ± 5.33 (70)	7.5 ± 5.36 (79)	−1.1 ± 5.8 (*N* = 36)	0.079
95% CI of mean	(6.65, 10.06)	(7.5, 10.25)	(5.70, 8.24)	(6.32, 8.72)	(−3.1, 0.9)	
KOOS—symptoms/stiffness						
Mean ± SD (*N*)	62.0 ± 20.1 (34)	78.8 ± 14.6 (71)	83.5 ± 14.0 (71)	86.3 ± 12.6 (79)	24.6 ± 19 (*N* = 30)	<0.001
95% CI of mean	(54.9, 69.0)	(75.4, 82.3)	(80.2, 86.8)	(83.5, 89.1)	(17.5, 31.8)	
KOOS—pain						
Mean ± SD (*N*)	69.2 ± 18.7 (34)	86.7 ± 10.8 (69)	91.7 ± 9.2 (71)	92.7 ± 8.6 (79)	22.2 ± 13.7 (*N* = 30)	<0.001
95% CI of mean	(62.7, 75.8)	(84.1, 89.3)	(89.5, 93.9)	(90.8, 94.7)	(17.1, 27.3)	
KOOS—function: daily living						
Mean ± SD (*N*)	73.5 ± 19.2 (34)	91.8 ± 10.8 (71)	95.6 ± 7.1 (71)	96.7 ± 6.0 (79)	21.2 ± 16.1 (*N* = 30)	<0.001
95% CI of mean	(66.8, 80.2)	(89.2, 94.3)	(93.9, 97.2)	(95.3, 98.0)	(15.2, 27.2)	
KOOS—function: sports and recreational activities						
Mean ± SD (*N*)	29.8 ± 31.3 (31)	63.9 ± 27.4 (67)	79.1 ± 19.9 (71)	82.0 + 18.5 (79)	50.2 ± 31.7 (*N* = 27)	<0.001
95% CI of mean	(18.3, 41.3)	(57.2, 70.6)	(74.4, 83.8)	(77.9, 86.2)	(37.7, 62.8)	
KOOS—quality of life						
Mean ± SD (*N*)	25.9 ± 19.9 (34)	53.0 ± 20.1 (71)	64.5 ± 20.3 (71)	70.9 ± 18.9 (79)	47.0 ± 22 (*N* = 30)	<0.001
95% CI of mean	(19.0, 32.9)	(48.2, 57.7)	(59.7, 69.3)	(66.7, 75.2)	(38.8, 55.2)	
Return to sports index						
Mean ± SD (*N*)	NA	50.2 ± 26.39 (59)	58.8 ± 25.04 (70)	66.6 ± 24.06 (73)	NA	<0.001
95% CI of mean		(43.3, 57.1)	(52.9, 64.8)	(61.0, 72.2)		
Current status—return to play						
Percentage (*N*)	NA	35.1% (20/57)	61.4% (43/70)	68% (51/75)		

Abbreviations: ANOVA, analysis of variance; CI, confidence interval; IKDC, International Knee Documentation Committee; KOOS, Knee injury and Osteoarthritis Outcome Score; SD, standard deviation.

## DISCUSSION

The most important finding of this study was a zero percent retear rate during the first postoperative year after BEAR, with low rate of adverse events even with 61% returning to sport at 9 months. In this cohort study of the first 100 subjects undergoing the BEAR procedure in the Bridge registry, there was 95% subject retention at 1 year with low reinjury rate and reoperation rate at 1 year. Two ACL retears occurred among the 31 subjects with two 2‐year follow‐up. This study also showed significant improvement of patient‐reported outcomes for knee function, pain and stability 1 year after the BEAR procedure, with the exception of Marx activity score. Additionally, instability examination over time demonstrated excellent stability in the majority of subjects.

This is the first study of real‐world outcomes of the BEAR procedure after commercial release. Earlier trials including the feasibility trial and the subsequent BEAR II randomised prospective study, were constrained by inclusion criteria, especially time from injury to surgery less than 45 days, as well as age as the studies were primarily performed at a single centre with younger patients (Boston Children's Hospital) [11, 12]. The Bridge registry includes a patient population that has a broader range of time from injury to surgery and older patients with lower Marx activity scores than prior BEAR studies. The overall difference in demographics would suggest a selection bias for surgical indications for the BEAR procedure in the Bridge registry. The Bridge registry population may better reflect the broader postcommercialisation use of BEAR, with over 4000 BEAR procedures having been performed at the time of writing of this manuscript.

Given the older population in the Bridge registry, and the known 0% reinjury rate at 2 years in prior BEAR studies in subjects over the age of 22 as well as low rates of reinjury at 2 years in other studies of ACL repair in subjects over the age of 21, we would expect to see low rates of ACL reinjury in this cohort [22, 24]. The mean age in the Bridge registry was 31.3 years and only 25% were under the age of 18. By contrast in BEAR II, the mean age of the BEAR patients was 17 [11]. A low retear rate is expected based on higher average age in this cohort, however, there is also value in collecting data on individuals outside the age minimum in prior studies, albeit a small part of the subject population. Despite the average older age of subjects, the Bridge registry does have a broader age range of enrolled subjects, with a minimum age of 7 years old. The inclusion of younger subjects within the registry, outside the range of enrollment criteria of FDA trials, will provide additional knowledge with longer‐term follow‐up regarding potential benefits to the paediatric population, including preadolescents not studied previously.

A 2025 systematic review and meta‐analysis that included studies comparing primary ACL repair or BEAR to ACL reconstruction demonstrated a relative risk of revision ACL surgery for primary repair of 4.77 and 2.42 for BEAR as compared to ACL reconstruction, suggesting a more favourable outcome of BEAR than isolated primary repair of the ACL [22]. Direct comparison of primary ACL repair to BEAR is, however lacking in the literature. The same systematic review showed no differences in outcomes between primary repair and reconstruction in subjects greater than 21 years of age, with 2.3% rate of revision surgery in that subgroup. The BEAR procedure has been shown to have a 0% rate of revision surgery in subjects over the age of 22 [24]. Retear rates and rates of ACL revision surgery in the current study cannot be compared to these results given lack of two 2‐year follow‐up.

Recognising the older age and lower MARX scores in the Bridge registry cohort as compared to prior BEAR studies, the study population within the registry did have early self‐reported return to sports with 61% reporting return to sport at 9 months. This analysis is focused on the first 100 subjects with 1 year of follow‐up, thus a significant portion of the population was participating in sport during the last 3 months of the year. No retears occurred during the first year, while two did occur between 1 and 2 years. A significant limitation of this interim analysis study is the length of follow‐up limiting risk of reinjury with further exposure to at risk activities over time.

Of additional interest in this cohort is the introduction of modifications to the classic technique for performing the BEAR procedure. No significant differences in adverse events were identified between the two procedure groups, but significant differences in age and sex were noted, such that the modified technique group was noted to be older and include more women. The modified techniques also included a range of variations thus are a heterogeneous group of modified techniques with the most common modification being use of a suture anchor rather than a button on the femur and use of other nonabsorbable, stronger bracing suture rather than ethibond through the BEAR implant. Theoretical risk of these modifications could be loss of motion or stiffness; however, the modified group did not show any increased risk of need for manipulation and achieved range of motion including passive hyperextension and passive flexion motion of 0.6 ± 2.9 degrees and 135.9 ± 5.5 degrees, respectively. The classic technique group in the registry achieved passive hyperextension of 2.8 ± 4.9 degrees and passive flexion of 136.0 ± 26.7 degrees at 12 months. More data are needed to fully understand the impact on range of motion, stability, and outcomes of the BEAR procedure with modifications. Studies with standardisation of the modifications may shed further light on this topic. Additionally, a common adverse event in the classic technique group was return to OR for retained Ethibond suture in the notch. This was not a common adverse event in previous trials, but is worthy of study in future comparisons of classic versus modified techniques.

Another weakness of this study is that among the first 100 subjects in the Bridge registry, the majority were enrolled after the surgery, such that there were low numbers of preoperative surveys and inconsistencies in completeness of documentation of preoperative examinations. Regardless, among those with pre and postoperative values, all PROs aside from MARX significantly improved over time. Excellent KOOS and IKDC scores were reported at 12 months. These improvements are clinically significant and meaningful, with all mean preoperative values falling below thresholds associated with a patient accepting symptom state (PASS) for ACL injury, and all 12‐month postoperative mean values exceeding the threshold for PASS [29]. Additionally, excellent stability testing outcomes including Lachman's and pivot shift findings were noted. An additional limitation of this study is that in‐person 12‐month stability testing was available in only 75 of the 100 subjects in this cohort, allowing for the potential of some subjects to report no reinjury on phone call and survey follow‐up, but potentially develop asymptomatic increased laxity over time.

Lastly, without prolonged exposure to risk with longer‐term follow‐up and exposure to sport, the risk of retear, reinjury and reoperation is likely underestimated, and longer‐term follow‐up is needed. While low rates of reinjury are seen thus far at 1 year and with early return to play in this cohort, further research is warranted to better understand long‐term outcomes of BEAR with and without modified techniques and with more widespread utilization and indications.

## CONCLUSIONS

The rates of adverse events after BEAR with and without modification are low, with excellent stability and ROM attained at 1 year with and without modified techniques. While early clinical results of postcommercialisation use of BEAR are promising, further research is warranted to better understand long‐term outcomes with and without technique modifications.

## AUTHOR CONTRIBUTIONS

Dr. Jocelyn Wittstein is the primary writer, but all authors have contributed to data collection, review, analysis and edits of the manuscript.

## CONFLICT OF INTEREST STATEMENT

All the authors have received funding/sponsorship for this study from Miach and have served on the Miach publication committee and all are affiliated with Miach which has proprietary rights to the BEAR implant. Brian Lau: Miach consultant, Cytek‐ consulatant, Convergence‐ consultant, NewClip‐ consultant. Daryl Osbahr: Miach consultant, paid speaker. Dennis Kramer: Miach consultant. Jocelyn Wittstein: Miach consultant, lead writer; ViewFi stock options, advisory board; Arthrex paid speaker; Vericel paid speaker. Jacqueline Brady: Miach consultant, study lead. Sabrina Strickland: Miach consultant, study lead; Vericel paid speaker. Andreas Gomoll: consultant Smith and Nephew, Vericel, Bioventus. Seth Sherman: consultant Linvatech, Arthrex, Vericel, Bioventus, Synthes, Smith and Nephew. Dean Taylor: consultant depuy synthes. Sean Keyes: Miach consultant. Alexander K. Meininger: Miach consultant. Marc Pietropaoli: Miach consultant. Sean McMillan: consultant Miach, Linvatech, Medical Device Business Services, Anika Therapeutics, Trice Medical, Davol Inc, Pacira Pharmaceuticals.

## ETHICS STATEMENT

Subject Signature on Page 7 of the consent form indicates they have read and understand the consent form. They have been provided all information, all questions were addressed to their satisfaction and they agree to everything explained in the consent form. WCG/Connexus IRB (IRB Tracking ID: 20222900).

## Data Availability

The data that support the findings of this study are available from the corresponding author upon reasonable request.
